# Supplementary material for: Green Synthesis of Silver Nanoparticles Using Randia aculeata L. Cell Culture Extracts, Characterization, and Evaluation of Antibacterial and Antiproliferative Activity
Source: Nanomaterials (Basel). 2022 Nov 25;12(23):4184. doi: 10.3390/nano12234184 (PMC9736092; doi:10.3390/nano12234184)
Supplement: Supplementary file 1 [file nanomaterials-12-04184-s001.zip › nanomaterials-2008578-supplementary.pdf]

Supplementary Materials

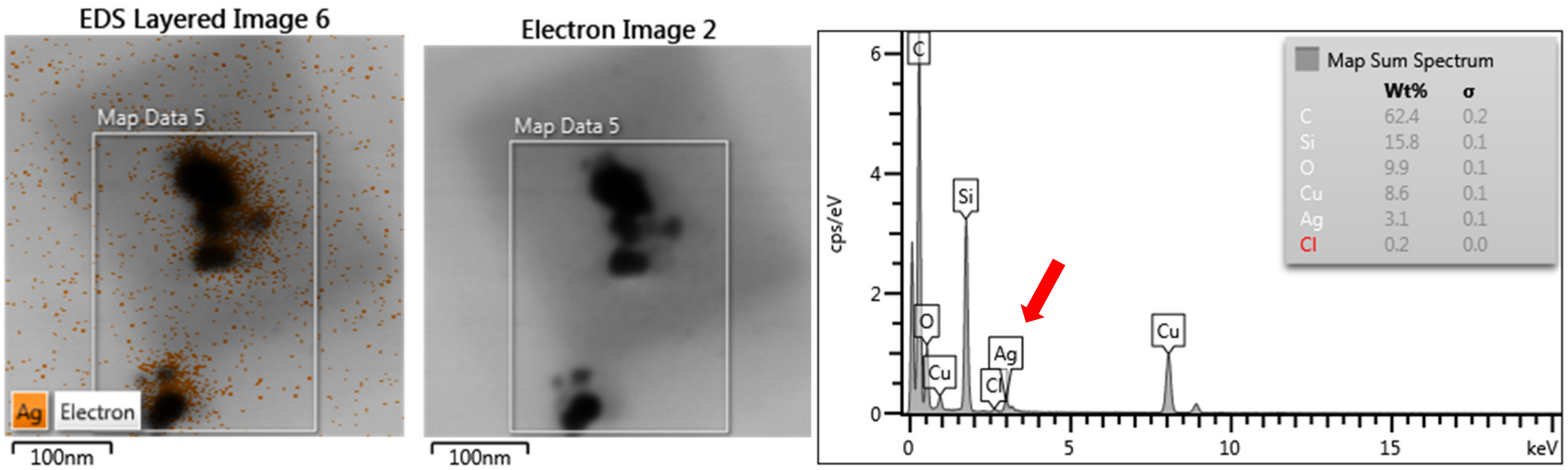

Label:	Map Sum Spectrum
Element List Type:	Current Spectrum
Processing Option:	All Elements
Ratio Standard Element:	Silicon
Ratio Standard Line:	K series
Specimen Thickness:	1 nm
Specimen Density:	0 g/cm^3
Automatic Line Selection:	Enabled
Thresholding:	Disabled
Deconvolution Elements:	None
Pulse Pile Up Correction:	Succeeded
Detector file:	X-Max 8
Efficiency:	File based

Figure S1. Elemental analysis by EDX of a sample of AgNPs indicating the presence of silver and other elements corresponding to the composition of the grid and the sample holder of the TEM system.

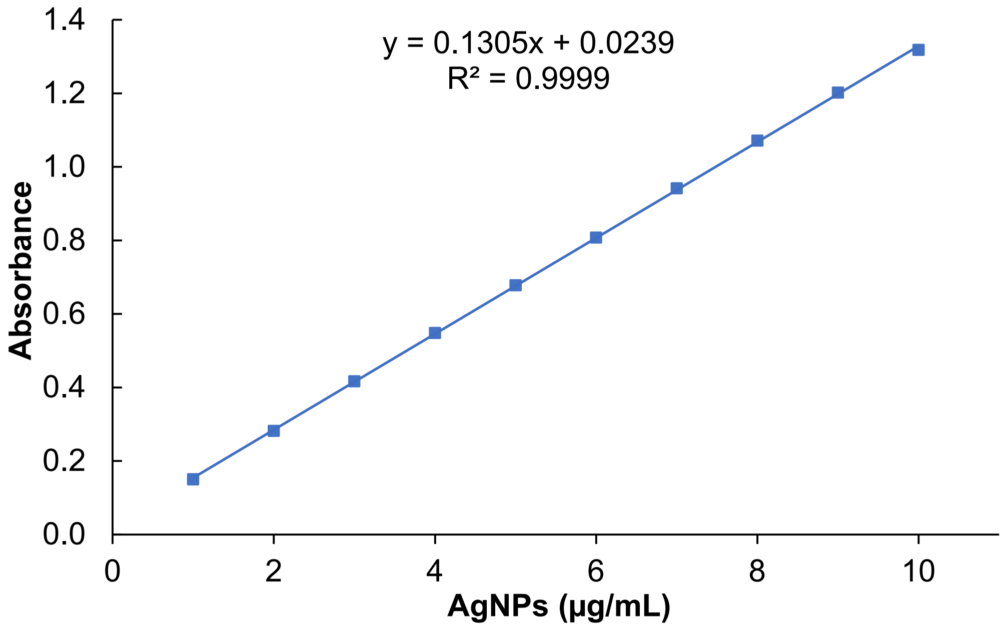

Figure S2. Calibration curve with 10 nm commercial AgNPs at an absorbance of 395 nm using a UV–Vis spectrophotometer.

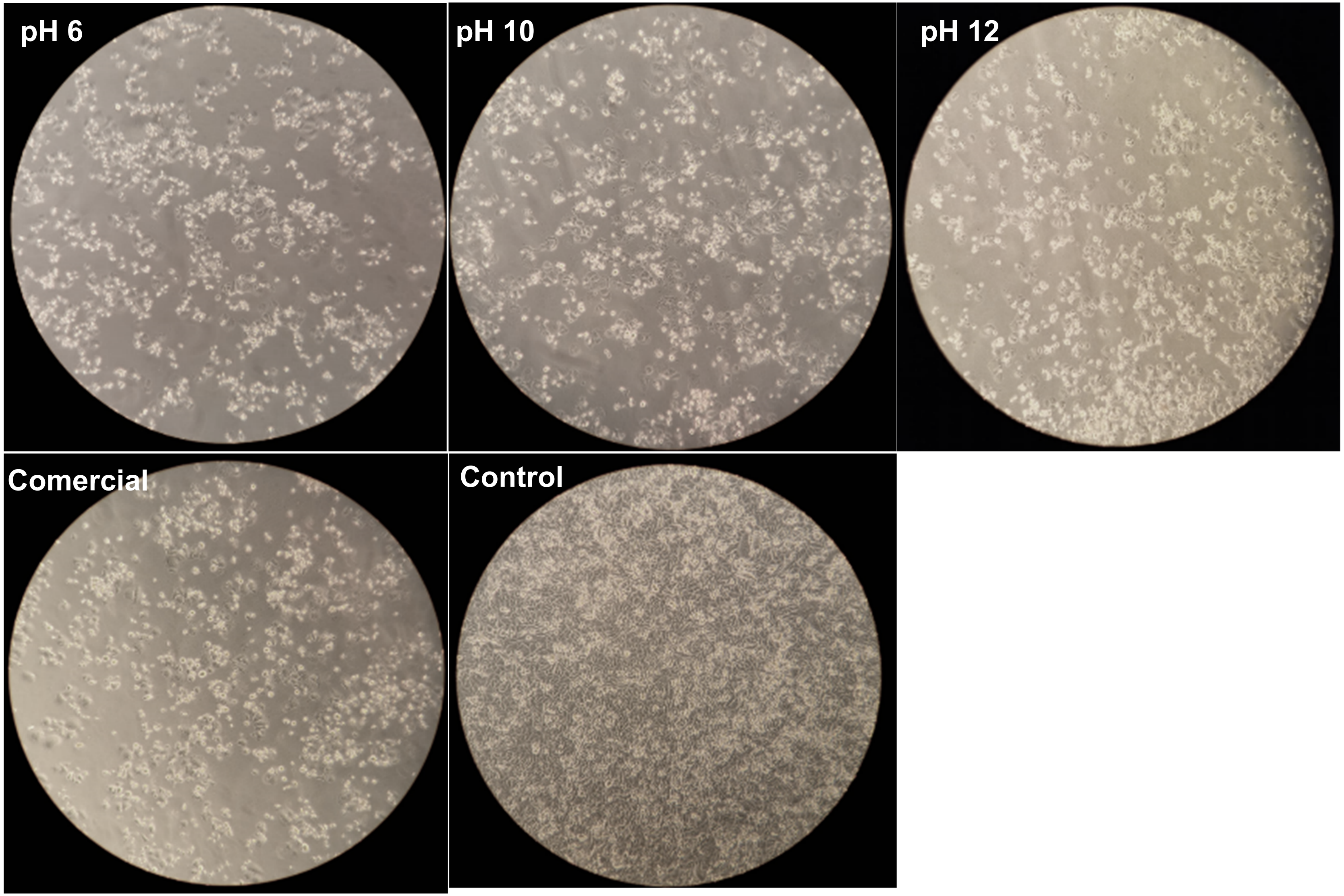

Figure S3. Microscopic images of the effect of AgNPs obtained at different pH and evaluated in the Hep3B cell line compared with commercial AgNPs (Sigma) and a control (DMSO).
